# Draft genome for *Flavobacterium psychrophilum* isolates from diseased coho salmon (*Oncorhynchus kisutch*) in Chile

**DOI:** 10.1128/mra.00547-24

**Published:** 2024-08-20

**Authors:** Ruben Avendaño-Herrera, Mónica Saldarriaga-Córdoba, Pedro Ilardi

**Affiliations:** 1 Universidad Andrés Bello, Laboratorio de Patología de Organismos Acuáticos y Biotecnología Acuícola, Facultad de Ciencias de la Vida, Viña del Mar, Chile; 2 Centro FONDAP, Interdisciplinary Center for Aquaculture Research (INCAR), Universidad Andrés Bello, Viña del Mar, Chile; 3 Centro de Investigación Marina Quintay (CIMARQ), Universidad Andrés Bello, Quintay, Chile; 4 Escuela de Medicina Veterinaria & Centro de Investigación en Recursos Naturales y Sustentabilidad, Universidad Bernardo O'Higgins, Santiago, Chile; 5 Depto. Investigación y Desarrollo, Farmacología en Aquacultura Veterinaria FAV S.A., Santiago, Chile; University of Southern California, Los Angeles, California, USA

**Keywords:** *Flavobacterium psychrophilum*, coho salmon, BCWD, RTFS

## Abstract

We present the draft genome sequences of six *Flavobacterium psychrophilum* isolates recovered from diseased coho salmon (*Oncorhynchus kisutch*) cultured by two farms in Chile. This study provides the first detailed insights into the genomic characteristics of this fish pathogen recovered from a host with limited information and cultured in Chile.

## ANNOUNCEMENT

*Flavobacterium psychrophilum*, a Gram-negative, filamentous, psychotropic bacterium, causes bacterial cold-water disease and rainbow trout fry syndrome in salmonids worldwide (1). Genetic studies suggest some specific genotypes for certain fish species, especially coho salmon (*Oncorhynchus kisutch*) (2, 3). To date, analyzed isolates from this fish species are limited, and none are from Chile. Herein, we present the draft genome sequences of six isolates recovered from diseased coho salmon cultured by two farms in Los Lagos Region (Chile).

Samples for bacterial isolation were taken from external lesions, the spine, the kidney, and the spleen of each coho salmon and streaked onto the tryptone yeast extract salts (TYES) medium (4). Colonies were recovered after incubation at 15°C for 7 days and streaked onto fresh TYES plates to isolate pure cultures. Six isolates were confirmed as *F. psychrophilum* via PCR analysis, following the protocol by Urdaci et al. (5), with genomic DNA extraction using the InstaGene matrix (Bio-Rad). All *F. psychrophilum* cultured underwent no more than two rounds of culturing and were stored at −80°C in CryoBank vials (Mast Diagnostica, Reinfield, Germany).

For genomic sequencing, three pure colonies of each *F. psychrophilum* were subjected to DNA extraction and were sent for genome sequencing to SeqCenter (Pennsylvania). Libraries were prepared using the Illumina DNA Prep Kit and IDT 10 bp UDI indices and sequenced on Illumina NextSeq 2000, producing 2 × 151-bp reads. Before *de novo* genome assembly, raw Illumina sequence data were quality-checked with FastQC v.0.12.1 (https://www.bioinformatics.babraham.ac.uk/projects/fastqc/). Next-generation sequencing reads were pre-processed with Geneious Prime v.2024.0.7 (www.geneious.com) with the following workflow: (i) pairing reads using the “set paired reads” function (insert size = 350  bp); (ii) trimming poor-quality bases from read ends using the BBDuk Trimmer plugin version 38.84 (6) (reads of <20 bp, and those with a quality score <20 were removed), with paired-read overlaps trimmed to ensure complete adapter removal; (iii) normalizing coverage by down-sampling reads in high-depth genome areas with the “error correct” and “normalize reads” functions using BBNorm version 38.84 (6); and (iv) removing duplicate reads using the Dedupe plugin. For the assembling process, Spades v3.13.0 was used (7), evaluating the quality and completeness of the final assemblies using CheckM v1.2.2 (8), and quality was checked by QUAST v.5.2.0 (9). The assembled genomes were then annotated using the RAST server v.2.0 (10). Default parameters were used for all software. Statistics of assembled genomes are shown in Table 1.

**TABLE 1:** Listing of *Flavobacterium psychrophilum* isolate genomes released to NCBI

Strain/library	Sampling tissue	SRA	Assembly	WGS	Total length^*a*^	Genome coverage	No. reads^*b*^	Contigs^*c*^	%GC^*b*^	N50 kb^*b*^	Genes^*a*^	Protein-coding^*a*^	tRNA^*a*^
Flp-133/S74	Kidney	SRR28673983	GCF_038405635.1	JBCARS01	2,694,494	100×	1,408,614	50	32.5	119.3	2,427	2,363	41
Flp-134/S78	External lesions	SRR28673979	GCF_038405535.1	JBCARW01	2,736,257	100×	1,693,310	117	32.5	55.3	2,472	2,408	39
Flp-138/S75	Kidney	SRR28673982	GCF_038405615.1	JBCART01	2,733,480	100×	1,466,238	119	32.5	55.3	2,471	2,407	39
Flp-142/S76	Kidney	SRR28673981	GCF_038405595.1	JBCARU01	2,694,456	100×	1,520,966	54	32.5	119.4	2,430	2,367	40
Flp-143/S77	Spleen	SRR28673980	GCF_038405575.1	JBCARV01	2,696,331	100×	2,048,008	52	32.5	119.3	2,431	2,369	39
Flp-146/S215	Spine	SRR28673984	GCF_038405655.1	JBCARR01	2,694,356	100×	4,004,454	61	32.5	98	2,433	2,371	39

^*a*^ Determined using RAST.

^*b*^ Normalized read depth using BBNorm.

^*c*^ Determined using Quast.

Genome assemblies ranged from 50 to 119 contigs and 2,694,356 to 2,736,257 bp with a G+C content of 32.5% (Table 1). To trace any plasmid, the filtered reads were mapped using SOAP (https://ccb-microbe.cs.uni-saarland.de/plsdb/) to the bacterial plasmid database (11). Only *F. psychrophilum* Flp-134 presented a 2,191-bp contig matching a plasmid (JBCARW000000000.1).

The *in silico* genome analysis discovered genes associated with secretion systems, including the type I and type IX secretion systems (T1SS and T9SS, respectively), as detected by the TXSScan:MascSyFinder-based detection of protein secretion systems (12). Genes associated with antibiotic resistance were not found in the Resistance Gene Identifier incorporated with the Comprehensive Antibiotic Resistance Database (https://card.mcmaster.ca/analyze/rgi). The present study provides initial comprehensive insights into the genomic characteristics of *F. psychrophilum* isolates from coho salmon farmed in Chile, with further details on virulence potential slated for future publication.

## Data Availability

This whole-genome shotgun project has been deposited in DDBJ/ENA/GenBank under the Accession Numbers JBCARR000000000-JBCARW000000000. The version described in this paper is version JBCARR-W010000000. BioProject ID PRJNA1099911 is publicly available and contains BioSample SAMN40951232-SAMN40951237. SRA records will be accessible with the following link after the indicated release date: https://www.ncbi.nlm.nih.gov/sra/PRJNA1099911. Genome annotations are available in https://doi.org/10.6084/m9.figshare.25979599.
